# Associations of cerebrospinal fluid measures of synaptic function with white matter microstructure and cognition in older adults

**DOI:** 10.3389/fnagi.2026.1851829

**Published:** 2026-06-15

**Authors:** Elizabeth R. Paitel, Corinne Pettigrew, Abhay Moghekar, Michael I. Miller, Andreia V. Faria, Marilyn Albert, Chan Hyun Na, Paul Worley, Anja Soldan

**Affiliations:** 1 Department of Neurology, Johns Hopkins School of Medicine, Baltimore, MD, United States; 2 Department of Biomedical Engineering, Johns Hopkins University, Baltimore, MD, United States; 3 Department of Radiology, Johns Hopkins University, Baltimore, MD, United States; 4 Department of Neuroscience, Johns Hopkins School of Medicine, Baltimore, MD, United States

**Keywords:** cognitive resilience, diffusion imaging, DTI, GluA4, NTPX2, synaptic, VGF

## Abstract

**Introduction:**

Lower levels of several synaptic proteins in cerebrospinal fluid (CSF) have been associated with greater cognitive decline among older adults, but there is limited understanding of their associations with brain structure. This study is among the first to examine the cross-sectional relationship between levels of three synaptic proteins (VGF, NPTX2, and GluA4) with magnetic resonance imaging (MRI) measures of white matter microstructure and volumes, and with cognitive performance. We also examined whether relationships between synaptic protein levels and white matter measures are influenced by CSF Alzheimer’s disease (AD) biomarker levels [ratio of p-tau_181_/(Aβ_42_/Aβ_40_)].

**Methods:**

Participants included 151 middle-aged and older adults without dementia (132 cognitively unimpaired, 19 mild cognitive impairment, *M*_age_ = 69.3 years). White matter volumes and microstructure [fractional anisotropy (FA), mean diffusivity (MD)], derived from MRI scans, were assessed in three regions: global cerebral, medial temporal lobe, and cerebellar peduncles.

**Results:**

In linear regression analyses, lower levels of NPTX2, VGF, and GluA4 were associated with lower FA and higher MD, even after accounting for CSF AD biomarker levels. Synaptic proteins were not associated with white matter volumes. Additionally, lower FA, higher MD, and lower VGF levels were associated with poorer executive function performance. An exploratory mediation analysis showed that cerebral white matter MD statistically mediated the relationship between VGF and executive performance.

**Discussion:**

These findings provide preliminary, cross-sectional support that VGF may act on cognition via white matter microstructure. Together the results suggest that white matter microstructure may represent one pathway linking synaptic proteins levels to cognitive performance among older adults. Future research is needed to advance understanding of the specific mechanisms driving these relationships.

## 1 Introduction

Synaptic transmission and white matter microstructure are two key features underlying efficient communication in the brain and are critical for cognitive performance (Kennedy, 2016; Morrison and Baxter, 2012; Moseley et al., 2002; Turken et al., 2008). Alterations in synaptic proteins are among the earliest brain changes associated with Alzheimer’s disease (AD; Ali et al., 2025; Van Der Ende et al., 2023), which is characterized by the abnormal accumulation of amyloid beta (Aβ) and tau proteins and subsequent neurodegeneration (for reviews see Camporesi et al., 2020; Chandra et al., 2019; Chen et al., 2023; Pelucchi et al., 2022). It has been suggested that levels of some synaptic markers are associated with cognitive resilience to AD-related brain changes, yet the mechanisms underlying such relationships are unclear (Colom-Cadena et al., 2020; de Vries et al., 2024; Taddei and Duff, 2025).

One synaptic marker studied in the context of cognitive resilience is the activity-dependent gene Neuronal Pentraxin 2 (NPTX2; Gómez de San José et al., 2022; Soldan et al., 2023; Xiao et al., 2017), which contributes to synapse formation and synaptic plasticity (Chang et al., 2010; Pelkey et al., 2015; Xiao et al., 2021). NPTX2 promotes clustering of postsynaptic glutamate AMPA receptors and thereby serves to strengthen excitatory synapses onto inhibitory interneurons (Pelkey et al., 2015). In AD brains, NPTX2 downregulation correlates with decreases in GluA4, a subunit of AMPA receptors, and the interaction of NPTX2 and GluA4 is crucial for maintaining the balance of excitation to inhibition in neural circuits (Xiao et al., 2017). A related synaptic protein that has been examined in relation to cognitive resilience is neurosecretory protein VGF (non-acronymic), a neuropeptide (Anderson et al., 2025; Quinn et al., 2021). Experimental studies indicate that VGF-derived peptides can modulate synaptic transmission and plasticity that are important for learning and memory, including long-term potentiation (Bozdagi et al., 2008; Hunsberger et al., 2007; Lin et al., 2015). Like NPTX2 and GluA4, VGF expression is reduced in AD brains (Beckmann et al., 2020) and VGF has been discussed as a potential target for AD (for reviews, see Askenazi et al., 2023; Beckmann et al., 2020; Quinn et al., 2021).

Prior work has shown that levels of NPTX2, GluA4, and VGF in cerebrospinal fluid (CSF) decline across the clinical spectrum of AD (Duits et al., 2018; Galasko et al., 2019; Libiger et al., 2021; Lleó et al., 2019; Lu et al., 2025; Nilsson et al., 2024; Soldan et al., 2019; Xiao et al., 2017). Lower CSF levels of these proteins are associated with greater cognitive decline and faster clinical progression in the symptomatic stages of AD (Duits et al., 2018; Galasko et al., 2019; Llano et al., 2019; Llano et al., 2023; Swanson and Willette, 2016; Xiao et al., 2017). Among individuals without cognitive impairment, lower CSF levels of NPTX2 are associated with faster progression from unimpaired cognition to MCI symptom onset (Soldan et al., 2023), and lower levels of NPTX2 and VGF are associated with poorer episodic memory (Kirsebom et al., 2025) and greater rates of cognitive decline (Anderson et al., 2025; Lu et al., 2025), with similar non-significant patterns reported with GluA4 (Anderson et al., 2025).

Despite the growing evidence linking these synaptic markers to cognitive outcomes, there is limited understanding of their impact on brain structure and function in humans. Prior neuroimaging work indicates that lower levels of NPTX2 are associated with greater longitudinal brain atrophy among participants with unimpaired cognition at baseline (Vazquez et al., 2025) and lower brain volumes and cortical thickness in participants spanning the AD continuum (Kirsebom et al., 2025; Nilsson et al., 2024; Swanson and Willette, 2016; Xiao et al., 2017). Similarly, lower baseline levels of VGF have been associated with greater hippocampal atrophy (Lu et al., 2025) and lower cortical thickness (Afxenti et al., 2025) in samples spanning the AD spectrum. Additionally, lower levels of NPTX2 were associated with lower resting state functional MRI (fMRI) connectivity in the salience/ventral attention network in participants without dementia (Soldan et al., 2019). However, to-date, no research to our knowledge has analyzed associations between these synaptic markers with measures of white matter microstructure and volume. Given prior studies have reported that these synaptic markers are related to gray matter structure (i.e., volumes, thickness), we hypothesized that they would also be associated with white matter structure.

The current study therefore examined the associations between CSF levels of the synaptic proteins NPTX2, VGF, and GluA4 with cerebral, medial temporal lobe (MTL), and cerebellar peduncle white matter microstructure metrics from diffusion tensor imaging (DTI) and with volumes of the same white matter regions. We hypothesized that lower levels of all three synaptic markers would be associated with lower fractional anisotropy (FA) and higher mean diffusivity (MD), considered to reflect poorer white matter microstructure (Bennett and Madden, 2014; Madden et al., 2012). We also assessed whether these relationships remained significant after adjusting for levels of CSF biomarkers of AD pathology (p-tau_181_ and Aβ_42_/Aβ_40_) and whether they were moderated by AD biomarker levels. Based on prior studies (Anderson et al., 2025; Soldan et al., 2023; Vazquez et al., 2025), we hypothesized that these associations would remain significant after accounting for CSF AD biomarker levels and would not be moderated by AD biomarker levels. Finally, we investigated relationships between cognitive performance with white matter microstructure and synaptic protein levels, anticipating that poorer cognitive performance would be associated with lower FA, higher MD, and lower levels of all three synaptic markers. We also hypothesized that relationships with white matter macrostructure (i.e., volumetrics) would be weaker than with microstructure, given recent findings that microstructure is more sensitive to age- and AD-related changes than macrostructure (Guerreri et al., 2019; Ibrahim and Bennett, 2023; Schilling et al., 2022).

## 2 Methods

### 2.1 Participants

Data for the current study were obtained from the ongoing prospective, longitudinal Biomarkers for Older Controls at Risk of Dementia (BIOCARD) study,^1^ which began in 1995 at the National Institute of Health (NIH; Albert et al., 2014). The study stopped in 2005 for administrative reasons and was re-established in 2009 at the Johns Hopkins University (JHU) School of Medicine. Both at the NIH and at JHU, comprehensive clinical and cognitive assessments have been completed annually. CSF and blood were collected every other year at the NIH, and blood has been collected annually at JHU. Since 2015, 3 T magnetic resonance imaging (MRI) scans, amyloid positron emission tomography (PET) scans, and CSF have been collected at JHU approximately every 2 years. Tau PET scans have been collected at JHU since 2021. The BIOCARD study was approved by the JHU Institutional Review Board and all participants provided written informed consent.

The current study included cross-sectional data from 151 middle-aged to older adult participants, with detailed clinical and cognitive assessments (132 cognitively unimpaired, 19 with MCI), who had their first 3 T MRI scan between 2015 and 2019 and CSF data collected within 15 months of the MRI scan.

### 2.2 Clinical and cognitive assessment

Comprehensive clinical evaluations and cognitive assessments were conducted at each annual visit, which included a battery of neuropsychological tests (see Albert et al., 2014 for details) and a semi-structured interview based on the Clinical Dementia Rating (CDR) scale (Morris, 1997). The JHU BIOCARD Clinical Core staff generates a consensus diagnosis annually using procedures comparable to those established by the National Institute on Aging (NIA) Alzheimer’s Disease Centers program. First, a syndromic diagnosis is generated based on the following data: (1) clinical information pertaining to the medical, neurological, and psychiatric status of the individual; (2) reports of cognitive changes by the individual and collateral source based on the CDR interview; and (3) cognitive test scores relative to prior performance and age-matched published norms. Syndromic diagnostic categories included: (1) cognitively unimpaired, (2) MCI, (3) impaired not MCI, and (4) dementia, and were based on criteria of the NIA/Alzheimer’s Association working groups for a diagnosis of MCI (Albert et al., 2013) and AD dementia (McKhann et al., 2011). An “impaired not MCI” diagnosis is given in the case of contrasting information from the CDR interview and cognitive test scores (i.e., participants or collateral source reported concerns for cognitive changes in daily life, but the cognitive testing did not show changes, or vice versa). Because participants who are “impaired not MCI” do not meet MCI criteria, they were included with the group of cognitively unimpaired participants, consistent with prior BIOCARD study publications (see Albert et al., 2014). Diagnostic status was coded as a dichotomous variable, identifying participants who were cognitively unimpaired and those with MCI.

Cognitive performance was assessed using two composite scores for the domains of executive functioning and episodic memory, given prior findings reporting associations between these domains and CSF levels of these synaptic proteins (Anderson et al., 2025; Kirsebom et al., 2025), as well as with white matter microstructure (Kennedy and Raz, 2009a; Sasson et al., 2012). These composite scores were derived from a confirmatory factor analysis of 12 neuropsychological test scores (for details, see Soldan et al., 2019). The executive function composite included digit span backwards from the Wechsler Memory Scale-Revised (WMS-R; Wechsler, 1987), Trail Making Test part B (Reitan, 1958), and the Digit Symbol Test from the Wechsler Adult Intelligence Scale-Revised (WAIS-R; Wechsler, 1981). The verbal episodic memory composite consisted of Wechsler Memory Scale-Revised (WMS-R; Wechsler, 1987) Logical Memory delayed recall, WMS-R Paired Associates immediate recall, and California Verbal Learning Test recall over trials 1–5 (Delis, 2000).

### 2.3 *APOE* genotype

*APOE* genotyping was conducted by the digestion of polymerase chain reaction-amplified genomic DNA using restriction endonucleases (carried out by Athena Diagnostic, Worcester, MA). *APOE* ε4 genetic status was coded dichotomously: participants carrying at least one ε4 allele were designated as ε4 carriers and those without an ε4 allele were classified as non-carriers. Participants with ε2/ε4 alleles were included in the *APOE* ε4 carrier group, given their risk for AD pathology is similar to that of ε4 carriers (ε3/ε4), rather than ε2 carriers (Goldberg et al., 2020).

### 2.4 Magnetic resonance imaging acquisition

MRI scans were acquired on a 3 T Phillips Achieva scanner (Eindhoven, The Netherlands). The multi-modal protocol included a magnetization-prepared rapid gradient echo (MPRAGE) scan used for anatomical reference and image registration (TR = 6.7 ms, TE = 3.1 ms, shot interval 3,000 ms, flip angle = 8°, FOV = 240 × 256 mm^2^, 170 slices with 1 × 1 × 1.2 mm^3^ voxels, and scan duration = 5 min 59 s). Diffusion-weighted images were acquired from two spin-echo sequences (TR = 7.5 s, TE = 75 ms, FOV = 260 × 260, slice thickness = 2.2 mm, flip angle = 90, *b*-value = 700, number of gradients = 33, axial plane).

### 2.5 Image processing

The diffusion-weighted images were automatically pre-processed and segmented using MRICloud (Mori et al., 2016)^2^. The tensor reconstruction and quality control followed the pipeline of DTIStudio (Jiang et al., 2006)^3^. MRICloud is a web-based platform that uses a fully automated multi-atlas image parcellation algorithm that combines the image transformation algorithm, Large Deformation Diffeometric Mapping, based on two complementary contrasts, b0 and FA (e.g., MD, RD, FA, and fiber orientation; Ceritoglu et al., 2009), and a likelihood fusion algorithm for DTI multiatlas mapping and parcellation (Tang et al., 2014). This generated 168 regions of interest (ROIs) based on JHU-MNI atlas (Oishi et al., 2009), from which DTI scalar metrics (3 eigenvalues) and volume of white matter tracts were extracted. Parcellations for each participant were visually inspected to ensure that the automated segmentation process yielded accurate delineations of the structures of interest. Tensor metrics were further thresholded to minimize contamination by other tissues, including cerebrospinal fluid partial volume effects (white matter threshold FA > 0.25). Structural MPRAGE MRI scans were also processed with MRICloud to obtain measures of total ventricular volume and total intracranial volume (ICV; i.e., total volume of tissues, ventricles, and sulci from the MPRAGE scans).

The microstructural analyses focused on FA and MD values for three composite measures that have been used in prior publications (Brichko et al., 2022; Paitel et al., 2025; Soldan et al., 2022). Individual tracts were first z-score normalized, then bilateral tracts were summed, and values were averaged across tracts within each composite: (1) global cerebral white matter, which was comprised of the fornix, cingulum, uncinate fasciculus, corona radiata, superior longitudinal fasciculus, inferior frontal occipital fasciculus, corpus callosum, and posterior thalamic radiation; (2) MTL white matter, which consisted of the hippocampal cingulum, fornix, and uncinate fasciculus; and (3) cerebellar peduncle white matter, which included the superior, middle, and inferior cerebellar peduncles. The tracts within the global composite were chosen *a priori*, as they provide reliable FA and MD values using the fully automated MRICloud platform across participants ranging in age and level of neurodegeneration.

White matter volume was assessed for the same tracts as were used for the calculation of the microstructure composite scores described above, based on the DTI segmentation. Volume composite scores were computed by summing values for (1) global cerebral, (2) MTL, and (3) cerebellar peduncle white matter volumes. Each volume composite was adjusted for total ICV using regression, and the standardized residuals were used for data analysis. These volume composites were investigated as outcome variables in analyses parallel to the microstructure models, and also included as covariates in sensitivity analyses to determine if associations with microstructural measures were independent of the volumes. Ventricular volume was similarly adjusted for ICV and used as a covariate in sensitivity analyses, as described below.

### 2.6 Cerebrospinal fluid biomarker measures

CSF was collected via lumbar puncture after an overnight fast; samples were aliquoted into polypropylene cryotubes and stored in a − 80 °C freezer. Samples were thawed for the first time to measure amyloid beta (Aβ_40_ and Aβ_42_), phosphorylated tau (p-tau_181_), and total tau using fully automated electrochemiluminescence assays (Lumipulse G1200 platform; Fujirebio Diagnostics, Inc.). A summary measure of AD pathology, computed as the ratio of p-tau_181_ to Aβ_42_/Aβ_40_, was used in these analyses (Greenberg et al., 2022). Note that we used the Aβ_42_/Aβ_40_ ratio instead of Aβ_42_ alone to adjust for individual differences in total Aβ production and reduce the impact of pre-analytic factors (e.g., Gervaise-Henry et al., 2017; Lewczuk et al., 2016).

Levels of NPTX2, GluA4, and VGF were measured using parallel reaction monitoring mass spectroscopy (PRM-MS; Na et al., 2020) after thawing the CSF samples a second time. Three NPTX2 peptides (WPVETCEER, TESTLNALLQR, and AAVLQLR), two VGF peptides (LLQQGLAQVEAGR and VGEEDEEAAEAEAEAEEAER), and three GluA4 peptides (FVIDCEIER, NTDQEYTAFR, and LQNILEQIVSVGK) were synthesized and labeled with stable isotopes on arginine residues. These were added to the CSF specimens for quantification. The relative abundance of peptides was measured by PRM-MS following enzymatic digestion of the CSF samples with endoproteinase Lys-C and trypsin. Skyline software was used to perform the quantification of relative peptide abundance (MacLean et al., 2010). As described previously (Soldan et al., 2023), the three NPTX2 peptides were highly correlated with one another (*r* = 0.89 to *r* = 0.94) and were therefore combined into a single NPTX2 composite measure by z-scoring and then averaging them. The three GluA4 peptides were also highly correlated (*r* = 0.90 to *r* = 0.94), as were the two VGF peptides (*r* = 0.94); the respective peptides for GluA4 and VGF were therefore combined to create composite scores using the same approach (Anderson et al., 2025). The NPTX2, GluA4, and VGF composite scores were then log-transformed to correct for skewness.

### 2.7 Statistical analysis

Multiple linear regression models were used to examine the associations of the three synaptic marker composite scores with white matter microstructure and volume. The primary models included age, sex, *APOE* ε4, diagnostic status, and synaptic marker composites. Outcomes included FA and MD and volume of the global, MTL, and cerebellar peduncle white matter composites, with separate models run for each MRI measure, as well as for each of the synaptic protein composites. To adjust for multiple comparisons, a False Discovery Rate (FDR) correction was applied; results of interest (i.e., synaptic markers) that remained significant at an FDR-adjusted *p* < 0.05 threshold are marked with an asterisk in the corresponding results tables, with the number of corrections indicated in the Table notes (Benjamini and Hochberg, 1995).

Next, for MRI measures showing significant associations with one or more synaptic markers, we assessed whether this relationship remained significant after adjusting for CSF AD biomarker levels and whether AD biomarker levels moderated the relationship, using a second set of regression models that included p-tau_181_/(A*β*_42_/A*β*_40_) and the synaptic marker*p-tau_181_/(A*β*_42_/A*β*_40_) interaction term as additional model predictors. A third set of regression models examined the associations between the MRI measures that were significantly associated with synaptic markers and synaptic protein composites with the two domain-specific cognitive composite scores (outcomes), covarying age, sex, years of education, and diagnostic status.

For cases in which the statistical prerequisites for mediation analysis were met – significant associations between synaptic marker levels, white matter MRI metrics, and cognitive performance—exploratory mediation analyses were conducted examining whether the MRI metric mediated the relationship between the synaptic marker and cognition. The PROCESS macro (version 4.2; Hayes, 2017) implemented in SPSS (version 29) was used to conduct mediation analyses with bias-corrected bootstrapping (5,000 permutations), with models adjusted for age, sex, education, diagnostic status, *APOE* ε4, and p-tau_181_/(A*β*_42_/A*β*_40_). Direct and indirect effects were estimated, and significance of the indirect effect was assumed if the bootstrapped 95% confidence interval did not include zero.

Several sensitivity analyses were conducted on the primary models. The first sensitivity analysis additionally covaried white matter volume to account for the potential influence of atrophy in each region. In these models, the regional volume measure reflected the volume of the same regions from which microstructure metrics were derived. A second sensitivity analysis examined whether results changed when covarying ventricular volume to further address potential CSF partial volume contamination of FA/MD metrics and measured CSF protein levels (Bergström et al., 2024). A third sensitivity analysis examined whether the primary patterns of results changed when excluding participants with a diagnosis of MCI. A fourth sensitivity analysis examined whether results changed when covarying a summary vascular risk score, given the influence of vascular health on white matter microstructure (e.g., Kennedy and Raz, 2009b; Williams et al., 2019).

## 3 Results

### 3.1 Descriptive statistics

Descriptive statistics for the 151 participants included in the analyses, as well as separately for the cognitively unimpaired participants and those with MCI, are shown in Table 1. Participants were 69 years of age, on average, and predominantly female (64%). On average, the MCI group had fewer years of education, lower cognitive scores, and lower synaptic marker levels.

**Table 1:** Descriptive statistics for total sample.

Participant characteristic	Total sample (*n* = 151)	Cognitively unimpaired (*n* = 132)	MCI (*n* = 19)	Group difference (*p* value)
Age	69.30 (8.50)	68.97 (8.39)	71.64 (9.13)	0.201
Education (years)	17.08 (2.36)	17.23 (2.26)^*^	16.00 (2.83)^*^	0.033
Sex (% female)	64%	64%	63%	0.916
*APOE* ε4 (% ε4+)	33%	33%	32%	0.879
MMSE	29.04 (1.20)	29.27 (0.94)^*^	27.42 (1.54)^*^	<0.001
Executive composite	0.08 (0.49)	0.17 (0.41)^*^	−0.52 (0.60)^*^	<0.001
Episodic composite	0.19 (0.50)	0.26 (0.45)^*^	−0.33 (0.53)^*^	<0.001
NPTX2	0.59 (0.44)	0.63 (0.45)^*^	0.32 (0.25)^*^	0.006
VGF	0.58 (0.47)	0.61 (0.48)^*^	0.37 (0.36)^*^	0.038
GluA4	0.58 (0.47)	0.61 (0.48)^*^	0.36 (0.33)^*^	0.026
p-tau_181_/(A*β*_42_/A*β*_40_)	669.30 (619.26)	676.08 (625.49)	622.91 (588.73)	0.728

^*^Significant group difference between cognitively unimpaired and MCI diagnostic groups, uncorrected *p* < 0.05. MMSE, Mini-Mental State Examination total score. Values indicate mean (SD) unless otherwise noted. Total sample for executive composite *n* = 149; total sample for episodic memory *n* = 150.

### 3.2 Associations of synaptic markers with white matter microstructure

**Figure 1:**
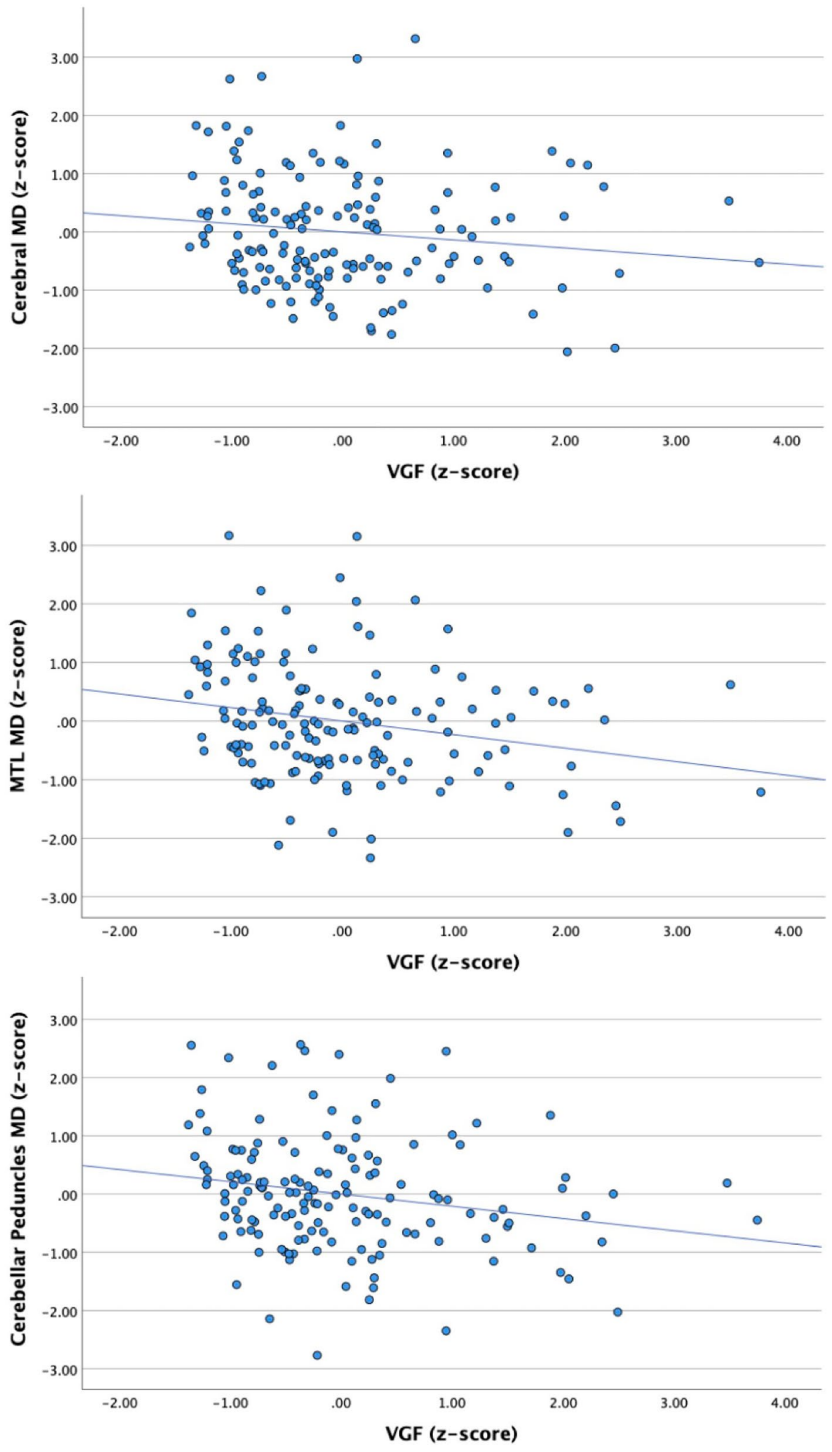
Lower levels of VGF were associated with higher mean diffusivity (MD; residualizing age, sex, APOE ε4, and diagnostic status) in three white matter composites: cerebral white matter (top), medial temporal lobe (MTL; middle), and cerebellar peduncles (bottom). Patterns were similar for NPTX2 and GluA4 (not shown).

In the primary regression models, lower levels of all three synaptic markers were associated with higher MD in all ROIs (*p*s < 0.05, except NPTX2 and cerebral MD, where *p* = 0.08; Table 2; Figure 1). Additionally, lower VGF and GluA4 were associated with lower FA in the cerebral and MTL ROIs, though these relationships were not significant following correction for multiple comparisons. Regarding covariates, older age was consistently associated with lower FA and higher MD in all models (*p*s < 0.01) except those with cerebellar FA. Women had lower MD in the cerebral (only in models with GluA4) and MTL composites (*p*s < 0.05). A diagnosis of MCI was associated with higher MD and lower FA (only in models with VGF and GluA4) in the MTL composite specifically (*p*s < 0.05). *APOE* ε4 was not a significant covariate in any of the models (*p*s ≥ 0.099).

**Table 2:** Results from multiple linear regression models assessing the associations of synaptic protein levels with white matter microstructure covarying age, sex, *APOE* ε4, and diagnostic status.

Cerebral FA	Coeff	SE	*t*	*p*	*f* ^2^	Cerebral MD	Coeff	SE	*t*	*p*	*f* ^2^
NPTX2	0.342	0.244	1.402	0.163	0.015	NPTX2	−0.409	0.233	−1.757	0.081	0.023
VGF	**0.547**	**0.226**	**2.415**	**0.017**	**0.040**	VGF	**−0.479**	**0.210**	**−2.275**	0.024^*^	**0.036**
GluA4	**0.470**	**0.232**	**2.026**	**0.045**	**0.005**	GluA4	**−0.450**	**0.215**	**−2.094**	0.038^*^	**0.001**

MTL FA	Coeff	SE	*t*	*p*	*f* ^2^	MTL MD	Coeff	SE	*t*	*p*	*f* ^2^
NPTX2	0.332	0.248	1.339	0.183	0.013	NPTX2	**−0.664**	**0.222**	**−2.994**	0.003^*^	**0.066**
VGF	**0.485**	**0.227**	**2.139**	**0.034**	**0.032**	VGF	**−0.672**	**0.200**	**−3.363**	<0.001^*^	**0.078**
GluA4	**0.519**	**0.231**	**2.249**	**0.026**	**0.008**	GluA4	**−0.686**	**0.203**	**−3.372**	**<0.001** ^*^	**0.029**

Cerebellar FA	Coeff	SE	*t*	*p*	*f* ^2^	Cerebellar MD	Coeff	SE	*t*	*p*	*f* ^2^
NPTX2	0.213	0.262	0.811	0.419	0.005	NPTX2	**−0.791**	**0.289**	**−2.736**	**0.007** ^*^	**0.055**
VGF	0.459	0.238	1.925	0.056	0.026	VGF	**−0.807**	**0.259**	**−3.119**	**0.002** ^*^	**0.067**
GluA4	0.364	0.244	1.493	0.138	0.008	GluA4	**−1.029**	**0.258**	**−3.983**	**<0.001** ^*^	**0.055**

**Bold**, un-corrected *p* < 0.05. ^*^Effect remains significant after applying FDR correction for multiple comparisons (nine comparisons, FDR-adjusted *p* value < 0.05). Cohen’s *f*^2^ effect size generally interpreted as small ≥ 0.02, medium ≥ 0.15. Coeff, coefficient (unstandardized); FA, fractional anisotropy; MD, mean diffusivity; MTL, medial temporal lobe; SE, standard error.

In the models that included CSF AD biomarkers, the interactions between the synaptic markers and p-tau_181_/(A*β*_42_/A*β*_40_) were non-significant (*p*s ≥ 0.129; data not shown). In the reduced models excluding the non-significant interaction terms, the patterns of results for white matter MD were largely unchanged from the primary models: lower levels of all three synaptic markers tended to be associated with higher MD in all ROIs (*p*s < 0.05, except NPTX2 and cerebral MD, where *p* = 0.09; Supplemental Table 1). Additionally, lower VGF was associated with lower FA in the cerebral ROI, and lower GluA4 was associated with lower FA in the MTL ROI, though these associations were not significant after correction for multiple comparisons. More abnormal AD biomarker levels were associated with lower MD in the cerebellar composite and higher FA in the MTL composite (*p*s < 0.05).

### 3.3 Associations of synaptic markers with white matter volumes

The synaptic markers were not significantly associated with white matter volumes (*p*s ≥ 0.088). The only exception was a positive association between VGF and MTL volume (*p* = 0.044), which did not survive multiple comparison correction (Table 3). Given the lack of significant associations with white matter volumes, follow-up models examining interactions between synaptic protein levels and AD biomarker levels were not run for the MRI volumes.

**Table 3:** Results from multiple linear regression models assessing the associations of white matter volumes and synaptic proteins, covarying age, sex, *APOE* ε4, and diagnostic status.

Cerebral WM volume	Coeff	SE	*t*	*p*	*f* ^2^
NPTX2	0.067	0.192	0.349	0.727	0.001
VGF	0.182	0.177	1.028	0.305	0.001
GluA4	0.276	0.178	1.554	0.123	0.017

MTL WM volume	Coeff	SE	*t*	*p*	*f* ^2^
NPTX2	0.292	0.195	1.499	0.136	0.017
VGF	**0.360**	**0.177**	**2.032**	**0.044**	**0.030**
GluA4	0.307	0.179	1.715	0.088	0.021

Cerebellar peduncle volume	Coeff	SE	*t*	*p*	*f* ^2^
NPTX2	−0.036	0.195	−0.182	0.856	0.000
VGF	−0.044	0.183	−0.244	0.808	0.000
GluA4	−0.010	0.184	−0.054	0.957	0.000

**Bold**, un-corrected *p* < 0.05. Coeff, coefficient (unstandardized); MTL, medial temporal lobe; SE, standard error; WM, white matter. Cohen’s *f*^2^ effect size generally interpreted as small ≥ 0.02, medium ≥ 0.15.

### 3.4 Results from sensitivity analyses

The first sensitivity analysis showed that associations between the CSF synaptic markers and the microstructural measures remained consistent when additionally covarying white matter volume to account for the potential influence of regional atrophy (Supplemental Table 2). The second sensitivity analysis showed that associations between the CSF synaptic markers and the microstructural measures were attenuated when covarying ventricular volume: only the negative relationships between GluA4 and MD in the MTL and cerebellar composites remained significant (Supplementary Table 3). A sensitivity analysis excluding the *n* = 19 participants with MCI showed that the associations between the synaptic markers with cerebral FA and MD and MTL FA were no longer significant, while associations between MTL and cerebellar MD with all three markers remained significant (Supplementary Table 4). A final sensitivity analysis showed that a summary vascular risk score was not a significant covariate in any models, and the relationships between synaptic markers and white matter microstructure were consistent with the primary models (Supplementary Table 6).

### 3.5 Relationships between synaptic markers, white matter microstructure, and cognitive performance

Higher MD and lower FA in the cerebral, MTL, and cerebellar composites were associated with poorer executive function performance (*p*s < 0.05, except for FA in the MTL, where *p* = 0.085; Table 4). Lower levels of VGF were also associated with poorer executive performance (*p* = 0.05; Table 4), though this relationship was not significant following correction for multiple comparisons. In contrast, neither the white matter microstructure measures nor synaptic markers were associated with episodic memory performance (all *p*s ≥ 0.151, data not shown). When participants with a diagnosis of MCI were excluded, the patterns were consistent, though the association between VGF and executive function was attenuated (*p* = 0.089, Supplementary Table 5).

**Table 4:** Results from multiple linear regression models assessing the associations of white matter microstructure and synaptic markers with executive function performance.

Executive function	Coeff	SE	*t*	*p*	*f* ^2^
Cerebral FA	**0.064**	**0.027**	**2.391**	**0.018** ^*^	**0.040**
Cerebral MD	**−0.074**	**0.029**	**−2.585**	**0.011** ^*^	**0.047**
MTL FA	0.047	0.027	1.736	0.085	0.021
MTL MD	**−0.063**	**0.030**	**−2.12**	**0.036** ^*^	**0.032**
Cerebellar FA	**0.062**	**0.025**	**2.431**	**0.016** ^*^	**0.041**
Cerebellar MD	**−0.049**	**0.023**	**−2.106**	**0.037** ^*^	**0.031**
NPTX2	0.061	0.039	1.546	0.125	0.018
VGF	**0.071**	**0.036**	**1.981**	**0.050**	**0.027**
GluA4	0.049	0.037	1.299	0.196	0.012

**Bold**, un-corrected *p* < 0.05. ^*^Effect remains significant after applying FDR correction for multiple comparisons (six comparisons, FDR-adjusted *p* value < 0.05). Models covaried age, sex, education, and diagnostic status. Cohen’s *f*^2^ effect size generally interpreted as small ≥ 0.02, medium ≥ 0.15. Coeff, coefficient (unstandardized); FA, fractional anisotropy; MD, mean diffusivity; MTL, medial temporal lobe; SE, standard error.

**Figure 2:**
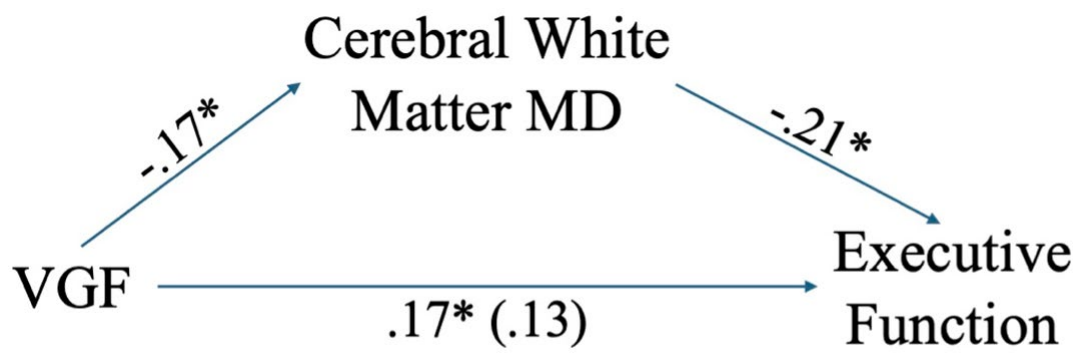
Mediation analysis showing that mean diffusivity (MD) in the cerebral white matter composite mediates the positive relationship between VGF and executive function performance. Model is adjusted for age, sex, education, diagnostic status, APOE ε4, and p-tau181/(Aβ42/Aβ40). Values show standardized beta coeficients, with value in parentheses showing the direct effect of VGF with executive function after accounting for cerebral white matter MD. *Effect is significant based on a bootstrap (5,000 permutations) approach and 95% confidence interval.

Given the associations of executive performance with both VGF and white matter microstructure, an exploratory mediation analysis was conducted, adjusting for age, sex, education, diagnostic status, *APOE* ε4, and p-tau_181_/(A*β*_42_/A*β*_40_). MD in the cerebral composite was selected for mediation, as it had the most robust association with cognitive performance. Cerebral MD was a significant mediator of the relationship between higher VGF and better executive performance (Figure 2; indirect effect: *β* = 0.037, 95% CI = 0.001–0.080; 22% of direct effect statistically mediated; direct effect of VGF with executive function: *β* = 0.166, 95% CI = 0.007 to 0.324, model *R*^2^ = 0.307; effect of VGF with cerebral MD 95% CI = −0.963 to −0.070, model *R*^2^ = 0.366; effect of cerebral MD with executive function, accounting for VGF 95% CI = −0.130 to −0.013, model *R*^2^ = 0.335; total effect model *p* < 0.001). Of note, the reverse was not significant—VGF did not statistically mediate the relationship between cerebral MD and executive function (indirect effect: *β* = −0.009, 95% CI = −0.030 to 0.002).

## 4 Discussion

The current study provides the first evidence to our knowledge of associations between lower levels of CSF synaptic proteins NPTX2, VGF, and GluA4 and poorer white matter microstructure (primarily lower MD, with weaker associations for FA). These relationships were evident in cerebral, MTL, and cerebellar regions in participants without dementia and remained significant after adjusting for CSF AD biomarker levels and vascular risk scores. The associations with DTI metrics were also independent of the volumes of the same white matter regions, which were not associated with synaptic marker levels. When excluding participants with MCI, only the MTL and cerebellar MD associations remained significant, which may indicate that these regions are more sensitive to synaptic and white matter microstructure alterations than a global measure of cerebral white matter in cognitively unimpaired participants. After accounting for ventricular volume, the relationships between synaptic proteins and white matter microstructure were attenuated, with only GluA4 remaining significant, highlighting the robustness of the GluA4 associations as well as the potential influences of ventricular enlargement, CSF dilution effects, or residual partial volume effects with NPTX2 and VGF. Additionally, poorer executive function performance was associated with lower FA, higher MD, and lower VGF levels. An exploratory mediation analysis indicated that cerebral white matter MD statistically mediated the relationship between VGF and executive performance. Taken together, these results provide preliminary, cross-sectional support for the view that levels of these specific synaptic proteins may be associated with executive function performance in dementia-free older adults through processes that are related to microstructural properties of white matter.

The present study expands on the limited existing research examining the associations between CSF synaptic markers, neuroimaging, and cognitive performance. Prior research in participants without dementia has largely focused on NPTX2, showing that lower levels of this protein are associated with greater longitudinal brain atrophy (Swanson and Willette, 2016; Vazquez et al., 2025) and with smaller brain volumes or cortical thickness in cross-sectional studies (Kirsebom et al., 2025; Nilsson et al., 2024; Xiao et al., 2017). Similarly, research integrating VGF has linked lower CSF levels among participants spanning the AD spectrum to greater longitudinal hippocampal atrophy (Lu et al., 2025) and lower cross-sectional cortical thickness (Afxenti et al., 2025). Lastly, a cross-sectional study reported a negative association between CSF NPTX2 levels with resting-state functional connectivity (Soldan et al., 2019). Taken together, these prior findings and the present ones suggest that lower levels of these specific synaptic proteins are associated with reduced integrity of global gray matter structure, poorer white matter microstructure, and alterations in large-scale functional networks among dementia-free older adults. We also expand on the existing literature by showing similar relationships with GluA4, which notably was the only protein to remain significantly associated with white matter microstructure after adjusting for ventricular volume. Future research on synaptic markers will benefit from inclusion of GluA4 as well as further investigation of the contributions of ventricular volume in analyses of CSF synaptic proteins (Bergström et al., 2024).

We also found that both the synaptic markers and white matter microstructure were associated with cognitive performance. Specifically, lower FA and higher MD were associated with poorer executive function performance, including when participants with a diagnosis of MCI were excluded from the analyses. This is consistent with many prior studies (Bettcher et al., 2016; Grieve et al., 2007; Kennedy and Raz, 2009a; Mayo et al., 2019). Lower VGF levels were also associated with poorer executive performance, and an exploratory mediation model showed that cerebral MD was a significant mediator of the relationship between lower VGF and poorer executive performance. This may indicate that white matter microstructure plays a role in the observed relationship between VGF and cognition, though future studies are needed to replicate these preliminary, cross-sectional findings. Unlike two prior longitudinal (Anderson et al., 2025) and cross sectional (Kirsebom et al., 2025) studies, in the current cross-sectional sample, episodic memory performance was not associated with synaptic marker levels. Nor was episodic memory associated with white matter microstructure metrics. These differences compared with prior studies are likely attributable to differences in sample characteristics and the cross-sectional nature of the current study. For example, Kirsebom et al. (2025) observed this association specifically within cognitively unimpaired participants who were Aβ+, while our analyses included all participants regardless of Aβ status. Future research is needed to replicate and further examine domain-specific associations with cognition. Notably, however, the current results support the view that synaptic degeneration is associated with cognition and suggests that this may be related to white matter microstructure alterations.

Associations between synaptic proteins and DTI metrics did not differ by AD biomarker levels, which is consistent with previous studies showing a lack of interactions between NPTX2, VGF, and GluA4 with AD biomarkers in relation to other cognitive and brain outcomes (Anderson et al., 2025; Soldan et al., 2023; Vazquez et al., 2025). For example, Soldan et al. (2023) found that baseline levels of NPTX2 improved prediction of MCI symptom onset after accounting for CSF AD biomarker levels, and NTPX2 did not interact with AD biomarkers, which they interpreted to suggest that both NPTX2 and AD biomarkers were independently associated with MCI symptom onset. This and the current findings are in line with the view that levels of these synaptic markers are not AD-specific, but are instead related to cognition across a variety of neurodegenerative disorders that are associated with synaptic dysfunction (Camporesi et al., 2020; Gómez de San José et al., 2022).

In this study, synaptic marker levels were not associated with white matter volumes. The exception was one association between VGF and MTL volume, which did not remain significant following adjustment for multiple comparisons. Thus, VGF, NPTX2, and GluA4 protein levels appear selectively associated with white matter microstructure, rather than with global white matter structure more broadly. This is consistent with and expands on prior studies showing that white matter microstructure may be a more sensitive measure than gross white matter volume, both in relation with cognition and age- and disease-related change (Guerreri et al., 2019; Ibrahim and Bennett, 2023; Schilling et al., 2022). These results raise the possibility that levels of these synaptic markers are also more strongly associated with gray matter microstructural properties than with global gray matter volumes, particularly among cognitively unimpaired individuals, given emerging evidence that alterations in gray matter microstructure may precede alterations in macrostructure in aging and neurodegeneration (Ibrahim and Bennett, 2023; Kantarci et al., 2005; Tang et al., 2024; Weston et al., 2015).

When excluding participants with MCI, associations between lower synaptic marker levels and lower microstructural integrity remained significant for the MTL and cerebellar white matter tracts, but not for the global cerebral composite, emphasizing the value of investigating regional composites. This may suggest that among cognitively unimpaired participants, the processes giving rise to associations between synaptic marker levels and microstructural properties are limited to MTL and cerebellar white matter, and that they become more widespread when participants experience clinically significant cognitive impairment. These findings are consistent with high expression of NPTX2 and GluA4 in the hippocampus (O'Brien et al., 2002; Xu et al., 2003), a region that is affected early in the course of AD, when individuals are still cognitively unimpaired (Soldan et al., 2015). Future research with larger samples spanning the AD continuum, as well as with a longitudinal study design, is necessary to understand the timing and sequence of changes in synaptic marker levels and regional white matter microstructure.

While the cerebellum is often excluded from neuroimaging analyses, our findings highlight the value of including this structure. GluA4 is most highly expressed in the cerebellum, followed by the brain stem and cerebral cortex (Kita et al., 2021; Thul and Lindskog, 2018). Cerebellar GluA4 has been shown to have a key role in the formation of associative memories in mice through its contributions to synaptic excitation at the cerebellar input layer (Kita et al., 2021). NPTX2 expression is also reported in the cerebellum (Gómez de San José et al., 2022), with NPTXs modulating long-term depression in cerebellar synapses (Cho et al., 2008). Further, VGF plays an important role in cerebellar development (Mizoguchi et al., 2019; Quinn et al., 2021), with smaller cerebellar volumes and motor coordination deficits in adulthood in VGF-overexpressing mice (Mizoguchi et al., 2019). The finding that CSF levels of these synaptic markers were associated with microstructure of the cerebellar peduncles, which connect the cerebellum to the cerebrum, may be relevant for understanding potential protective and resilience features of cerebellar connectivity with the cerebral cortex in the context of cognitive aging (Arleo et al., 2024; Bernard, 2022).

It is notable that the relationships between synaptic markers and white matter microstructure were stronger for MD compared to FA. This may be in part attributable to the sensitivity of FA to fiber orientation, with curving and overlapping fibers creating measurement challenges. FA tends to be high particularly in large myelinated fibers (e.g., corpus callosum), while MD is more sensitive to subtle alterations in microstructure, given its assessment of overall diffusion in all directions (Acosta-Cabronero et al., 2010; Assaf and Pasternak, 2008; Figley et al., 2022; Gold et al., 2012). Future studies incorporating multi-shell diffusion imaging protocols and multi-compartment modeling that have more biophysical specificity will be valuable for a more fine-grained understanding of potential mechanisms underlying the associations with synaptic markers.

The current study also found that in a sensitivity analysis that covaried total ventricular volume, associations of synaptic proteins with white matter microstructure were attenuated, with only GluA4 remaining significant. This may suggest that different proteins have differential sensitivity to dilution effects in CSF, related to larger ventricular size, with GluA4 potentially showing less sensitivity than NPTX2 and VGF (for similar findings, see Bergström et al., 2024). It has been proposed that this may be related to differences in regional expression patterns and sources of protein release into CSF (e.g., active secretion vs. leakage, Bergström et al., 2024). While processing of the diffusion tensor metrics in the current study included additional thresholding to minimize contamination by other tissues, including CSF partial volume effects, it is still possible that the attenuated associations following inclusion of ventricular volume are attributable to partial volume effects. Future studies are needed to explore potential mechanisms underlying differences in sensitivities to ventricular size of different synaptic proteins, and research with CSF proteins should include analysis of the contributions of ventricular volume on the relationships of interest.

While the current study cannot determine the mechanism by which synaptic markers and white matter microstructure may interact, all three proteins are involved in modulating inhibitory circuit function and circuit homeostasis (Bozdagi et al., 2008; Pelkey et al., 2015; Xiao et al., 2017). Loss of these proteins has been linked to hyperexcitability in animal models (Pelkey et al., 2015; Woo et al., 2024), which may contribute to downstream neurodegeneration. More broadly, this may suggest vulnerability of adaptive inhibitory circuit function in age-related neurodegenerative disease, including AD (Palop and Mucke, 2016; Verret et al., 2012; Xiao et al., 2017). It is possible that such synaptic dysfunction may lead to neuronal loss, contributing to degradation of white matter microstructure through Wallerian degeneration of projection axons and reduced axonal integrity. There may also be a role of synaptic glutamate activity in white matter more directly. Specifically, AMPA-mediated synaptic release of glutamate activates voltage-gated calcium channels that cluster together along axons, facilitating vesicular glutamate release in white matter. This process may be important for myelin maintenance, repair, and generation via communication with oligodendrocytes and oligodendrocyte precursor cells (Alix and de Jesus Domingues, 2011). Maintenance and repair of myelin via oligodendrocyte interaction is thus a potential mechanism underlying resilience in aging and AD (de Vries et al., 2024), but it necessitates future study with methods that can more directly assess such mechanisms. Ultimately, NPTX2, VGF, and GluA4 may be potential targets for prevention of and intervention with age-related cognitive decline, including in the context of various neurodegenerative diseases (Camporesi et al., 2020; de Vries et al., 2024; Taddei and Duff, 2025).

This study has several strengths, including a well-characterized cohort with comprehensive cognitive and clinical evaluations, as well as multiple CSF measures of synaptic proteins and AD biomarkers. Limitations of this study should be noted. The sample is primarily non-Hispanic White and highly educated. Selection biases related to demographic factors and health status of the sample may limit generalizability. Future studies with larger, more diverse, and community-based cohorts are needed to evaluate the generalizability of these results. Additionally, NPTX2 and VGF have been linked to psychiatric conditions, including anxiety and depression (e.g., Al Shweiki et al., 2020; Quinn et al., 2021), which merit future study in the context of cognitive aging and AD. Second, future analyses will benefit from a larger sample size spanning the preclinical to prodromal AD spectrum. Future analyses investigating microstructure of regionally specific cerebral white matter tracts may clarify patterns with synaptic proteins that were not observed using a global cerebral composite. Additionally, future studies with multi-shell diffusion imaging protocols and multi-compartment modeling will be important for further investigating the biophysical mechanisms underlying the observed relationships, given their ability to estimate different compartments of a diffusion voxel, including extracellular water and neurite density and orientation. Moreover, the current study is cross-sectional and provides preliminary evidence of the associations between synaptic proteins, white matter microstructure, and cognition. Importantly, a longitudinal design is essential for discerning differences in patterns related to the timing and progression of synaptic dysfunction and loss, neurodegeneration, AD pathology, cognition, and clinical symptoms.

## Data Availability

Publicly available datasets were analyzed in this study. This data can be found here: biocard-se.org.

## Footnotes

1
https://www.biocard-se.org

2
https://braingps.mricloud.org

3
www.MRIStudio.org
