# Supplementary material for: Associations of cerebrospinal fluid measures of synaptic function with white matter microstructure and cognition in older adults
Source: Front Aging Neurosci. 2026 Jun 15;18:1851829. doi: 10.3389/fnagi.2026.1851829 (PMC13311063; doi:10.3389/fnagi.2026.1851829)
Supplement: Supplementary file 1 [file Data_Sheet_1.docx]

*Associations of cerebrospinal fluid measures of synaptic function with white matter microstructure and cognition in older adults*

Supplemental Materials

**Associations between synaptic markers and white matter microstructure covarying CSF AD biomarker levels.**

In models additionally covarying an AD biomarker term as the ratio of p-tau_181_/(A*β*_42_/A*β*_40_), lower levels of all three synaptic markers were associated with higher MD in all ROIs, except NPTX2 and cerebral MD (Supplemental Table 1). Additionally, lower VGF was associated with lower FA in the cerebral ROI, and lower GluA4 was associated with lower FA in the MTL ROI. The relationships between cerebral MD with GluA4, as well as the associations with FA, did not remain significant following correction for multiple comparisons.

*Supplemental Table 1.* Results from multiple linear regression models assessing the associations of synaptic protein levels with white matter microstructure covarying age, sex, *APOE* ε4, diagnostic status, and p-tau_181_/(A*β*_42_/A*β*_40_).

| **Cerebral FA** | Coeff | *SE* | *t* | *p* | **Cerebral MD** | Coeff | *SE* | *t* | *p* |
| --- | --- | --- | --- | --- | --- | --- | --- | --- | --- |
| NPTX2 | 0.246 | 0.115 | 1.453 | 0.149 | NPTX2 | -0.410 | 0.239 | -1.716 | 0.088 |
| VGF | **0.512** | **0.233** | **2.195** | **0.030** | VGF | **-0.502** | **0.219** | **-2.289** | **0.024*** |
| GluA4 | 0.449 | 0.232 | 1.932 | 0.055 | GluA4 | **-0.440** | **0.218** | **-2.017** | **0.046** |
| **MTL FA** | Coeff | *SE* | *t* | *p* | **MTL MD** | Coeff | *SE* | *t* | *p* |
| NPTX2 | 0.294 | 0.251 | 1.170 | 0.244 | NPTX2 | **-0.624** | **0.228** | **-2.740** | **0.007*** |
| VGF | 0.366 | 0.235 | 1.561 | 0.121 | VGF | **-0.610** | **0.210** | **-2.903** | **0.004*** |
| GluA4 | **0.462** | **0.231** | **1.999** | **0.048** | GluA4 | **-0.643** | **0.207** | **-3.100** | **0.002*** |
| **Cerebellar FA** | Coeff | *SE* | *t* | *p* | **Cerebellar MD** | Coeff | *SE* | *t* | *p* |
| NPTX2 | 0.230 | 0.268 | 0.856 | 0.394 | NPTX2 | **-0.679** | **0.293** | **-2.318** | **0.022*** |
| VGF | 0.382 | 0.248 | 1.539 | 0.126 | VGF | **-0.615** | **0.267** | **-2.302** | **0.023*** |
| GluA4 | 0.349 | 0.247 | 1.417 | 0.159 | GluA4 | **-0.926** | **0.258** | **-3.587** | **<0.001*** |

Note. **Bold**, un-corrected *p* < .05. *Effect remains significant after applying FDR correction for multiple comparisons (nine comparisons, FDR-adjusted *p* value < 0.05). Coeff = coefficient; FA = fractional anisotropy; MD = mean diffusivity; MTL = medial temporal lobe; SE = standard error.

**Sensitivity analyses covarying white matter volume.**

The second set of sensitivity analyses included white matter volume composites as an additional model covariate to account for the potential influence of regional atrophy. In each model, the regional volume measure reflected the volume of the same regions from which microstructure metrics were derived. The relationships between synaptic markers and white matter microstructure were consistent with the primary models that did not covary white matter volume. Lower white matter volume was associated with higher MD in all three composites and with lower FA in the cerebral and MTL composites (*p*s < 0.01, data not shown).

*Supplemental Table 2.* Results from multiple linear regression models assessing the associations of synaptic protein levels with white matter microstructure covarying age, sex, *APOE* ε4, diagnostic status, and white matter volume

| **Cerebral FA** | Coeff | *SE* | *t* | *p* | **Cerebral MD** | Coeff | *SE* | *t* | *p* |
| --- | --- | --- | --- | --- | --- | --- | --- | --- | --- |
| NPTX2 | 0.330 | 0.231 | 1.427 | 0.156 | NPTX2 | -0.361 | 0.210 | -1.718 | 0.088 |
| VGF | **0.491** | **0.212** | **2.312** | **0.022** | VGF | -0.369 | 0.188 | -1.963 | 0.052 |
| GluA4 | 0.341 | 0.217 | 1.569 | 0.119 | GluA4 | -0.288 | 0.192 | -1.501 | 0.136 |
| **MTL FA** | Coeff | *SE* | *t* | *p* | **MTL MD** | Coeff | *SE* | *t* | *p* |
| NPTX2 | 0.239 | 0.250 | 0.955 | 0.341 | NPTX2 | **-0.571** | **0.224** | **-2.552** | **0.012** |
| VGF | 0.376 | 0.229 | 1.640 | 0.103 | VGF | **-0.554** | **0.203** | **-2.726** | **0.007** |
| GluA4 | 0.410 | 0.23 | 1.786 | 0.076 | GluA4 | **-0.590** | **0.203** | **-2.902** | **0.004** |
| **Cerebellar FA** | Coeff | *SE* | *t* | *p* | **Cerebellar MD** | Coeff | *SE* | *t* | *p* |
| NPTX2 | 0.224 | 0.264 | 0.851 | 0.397 | NPTX2 | **-0.806** | **0.287** | **-2.811** | **0.006** |
| VGF | **0.500** | **0.239** | **2.091** | **0.038** | VGF | **-0.840** | **0.256** | **-3.278** | **0.001** |
| GluA4 | 0.387 | 0.243 | 1.592 | 0.114 | GluA4 | **-1.032** | **0.253** | **-4.072** | **<0.001** |

Note. **Bold**, un-corrected *p* < .05. Coeff = coefficient; FA = fractional anisotropy; MD = mean diffusivity; MTL = medial temporal lobe; SE = standard error.

**Sensitivity analyses covarying total ventricular volume.**

The third set of sensitivity analyses examined whether covarying total ventricular volume impacted the associations between synaptic markers and white matter microstructure. While the directions of effects remain consistent with the primary models, the relationships between synaptic markers and white matter microstructure are attenuated with ventricular volume additionally covaried. Only the negative relationships between GluA4 and MD in the MTL and cerebellar composites remained significant. Greater ventricular volume was associated with higher MD in all three composites and with lower FA in the MTL and cerebellar composites.

*Supplemental Table 3.* Results from multiple linear regression models assessing the associations of synaptic protein levels with white matter microstructure covarying age, sex, *APOE* ε4, diagnostic status, and ventricular volume

| **Cerebral FA** | Coeff | *SE* | *t* | *p* | **Cerebral MD** | Coeff | *SE* | *t* | *p* |
| --- | --- | --- | --- | --- | --- | --- | --- | --- | --- |
| NPTX2 | 0.177 | 0.266 | 0.666 | 0.506 | NPTX2 | -0.129 | 0.250 | -0.516 | 0.607 |
| VGF | 0.432 | 0.248 | 1.745 | 0.083 | VGF | -0.245 | 0.227 | -1.080 | 0.282 |
| GluA4 | 0.315 | 0.251 | 1.257 | 0.211 | GluA4 | -0.219 | 0.229 | -0.958 | 0.340 |
| **MTL** **FA** | Coeff | *SE* | *t* | *p* | **MTL MD** | Coeff | *SE* | *t* | *p* |
| NPTX2 | 0.090 | 0.270 | 0.333 | 0.740 | NPTX2 | -0.372 | 0.238 | -1.565 | 0.120 |
| VGF | 0.271 | 0.247 | 1.100 | 0.273 | VGF | -0.400 | 0.215 | -1.862 | 0.065 |
| GluA4 | 0.295 | 0.249 | 1.188 | 0.237 | GluA4 | **-0.428** | **0.216** | **-1.983** | **0.049** |
| **Cerebellar FA** | Coeff | *SE* | *t* | *p* | **Cerebellar MD** | Coeff | *SE* | *t* | *p* |
| NPTX2 | -0.076 | 0.277 | -0.275 | 0.784 | NPTX2 | -0.465 | 0.312 | -1.489 | 0.139 |
| VGF | 0.254 | 0.254 | 1.002 | 0.318 | VGF | -0.514 | 0.279 | -1.843 | 0.067 |
| GluA4 | 0.125 | 0.256 | 0.488 | 0.627 | GluA4 | **-0.755** | **0.277** | **-2.725** | **0.007** |

Note. **Bold**, un-corrected *p* < .05. Coeff = coefficient; FA = fractional anisotropy; MD = mean diffusivity; MTL = medial temporal lobe; SE = standard error.

**Sensitivity analyses excluding participants with a diagnosis of mild cognitive impairment**

Sensitivity analyses were conducted excluding *n* = 19 participants with an MCI diagnosis, resulting in a sample of *n* = 132 participants. Supplemental Table 4 shows results from analyses evaluating the association between synaptic markers and white matter microstructure. Supplemental Table 5 shows results from models evaluating associations between DTI metrics and synaptic markers with executive function performance. The associations between synaptic markers and microstructure were restricted to MTL and cerebellar MD in this sub-sample, which may suggest that these regional composites and MD are more sensitive within a cognitively unimpaired sample.

*Supplemental Table 4.* Results from multiple linear regression models assessing the associations of synaptic protein levels with white matter microstructure covarying age, sex, regional volume, and *APOE* ε4, excluding participants with a diagnosis of MCI

| **Cerebral FA** | Coeff | *SE* | *t* | *p* | **Cerebral MD** | Coeff | *SE* | *t* | *p* |
| --- | --- | --- | --- | --- | --- | --- | --- | --- | --- |
| NPTX2 | 0.310 | 0.249 | 1.246 | 0.215 | NPTX2 | -0.367 | 0.241 | -1.526 | 0.130 |
| VGF | 0.375 | 0.232 | 1.615 | 0.109 | VGF | -0.368 | 0.219 | -1.680 | 0.095 |
| GluA4 | 0.356 | 0.236 | 1.509 | 0.134 | GluA4 | -0.317 | 0.223 | -1.419 | 0.158 |
| **MTL FA** | Coeff | *SE* | *t* | *p* | **MTL MD** | Coeff | *SE* | *t* | *p* |
| NPTX2 | 0.284 | 0.256 | 1.111 | 0.269 | NPTX2 | **-0.647** | **0.234** | **-2.762** | **0.007** |
| VGF | 0.321 | 0.235 | 1.364 | 0.175 | VGF | **-0.626** | **0.213** | **-2.944** | **0.004** |
| GluA4 | 0.452 | 0.237 | 1.904 | 0.059 | GluA4 | **-0.627** | **0.216** | **-2.9** | **0.004** |
| **Cerebellar FA** | Coeff | *SE* | *t* | *p* | **Cerebellar MD** | Coeff | *SE* | *t* | *p* |
| NPTX2 | 0.256 | 0.269 | 0.953 | 0.343 | NPTX2 | **-0.837** | **0.300** | **-2.787** | **0.006** |
| VGF | 0.443 | 0.249 | 1.782 | 0.077 | VGF | **-0.898** | **0.273** | **-3.292** | **0.001** |
| GluA4 | 0.373 | 0.254 | 1.471 | 0.144 | GluA4 | **-1.087** | **0.272** | **-4.004** | **<0.001** |

Note. **Bold**, un-corrected *p* < .05. Coeff = coefficient; FA = fractional anisotropy; MD = mean diffusivity; MTL = medial temporal lobe; SE = standard error.

*Supplemental Table 5.* Results from multiple linear regression models assessing the associations of white matter microstructure and synaptic proteins with executive function performance, covarying age, sex, and education, excluding participants with a diagnosis of MCI

| **Executive Function** | Coeff | *SE* | *t* | *p* |
| --- | --- | --- | --- | --- |
| Cerebral FA | **0.062** | **0.027** | **2.254** | **0.026** |
| Cerebral MD | **-0.083** | **0.029** | **-2.923** | **0.004** |
| MTL FA | 0.038 | 0.027 | 1.403 | 0.163 |
| MTL MD | **-0.062** | **0.029** | **-2.128** | **0.035** |
| Cerebellar FA | **0.058** | **0.026** | **2.262** | **0.025** |
| Cerebellar MD | **-0.045** | **0.023** | **-2.008** | **0.047** |
| NPTX2 | 0.052 | 0.037 | 1.384 | 0.169 |
| VGF | 0.059 | 0.035 | 1.716 | 0.089 |
| GluA4 | 0.049 | 0.036 | 1.356 | 0.177 |

Note. **Bold**, un-corrected *p* < .05. Coeff = coefficient; FA = fractional anisotropy; MD = mean diffusivity; MTL = medial temporal lobe; SE = standard error.

**Sensitivity analyses covarying vascular risk.**

Sensitivity analyses examined whether covarying vascular risk factors impacted the relationships between synaptic markers and white matter microstructure. These models included a composite vascular risk score as an additional model covariate. The composite vascular risk score reflected the sum of five dichotomous vascular risk factors, as previously published (Gottesman et al., 2017; Pettigrew et al., 2020; Soldan et al., 2020): hypertension, high cholesterol, diabetes, current smoking, and obesity (defined as body mass index>30). One participant was missing data for the vascular risk score; thus the sensitivity analyses included *n* = 150 participants. The summary vascular risk score was not a significant covariate in any models (all *p*s ≥0.116), and Supplemental Table 6 shows that the patterns of results were consistent with the primary models that did not covary vascular risk.

*Supplemental Table 6.* Results from multiple linear regression models assessing the associations of synaptic protein levels with white matter microstructure covarying age, sex, *APOE* ε4, diagnostic status, and vascular risk

| **Cerebral FA** | Coeff | *SE* | *t* | *p* | **Cerebral MD** | Coeff | *SE* | *t* | *p* |
| --- | --- | --- | --- | --- | --- | --- | --- | --- | --- |
| NPTX2 | 0.339 | 0.247 | 1.376 | 0.171 | NPTX2 | -0.410 | 0.235 | -1.742 | 0.084 |
| VGF | **0.547** | **0.228** | **2.395** | **0.018** | VGF | **-0.483** | **0.212** | **-2.279** | **0.024** |
| GluA4 | **0.474** | **0.234** | **2.026** | **0.045** | GluA4 | **-0.446** | **0.216** | **-2.062** | **0.041** |
| **MTL FA** | Coeff | *SE* | *t* | *p* | **MTL MD** | Coeff | *SE* | *t* | *p* |
| NPTX2 | 0.311 | 0.250 | 1.245 | 0.215 | NPTX2 | **-0.651** | **0.224** | **-2.909** | **0.004** |
| VGF | **0.477** | **0.229** | **2.086** | **0.039** | VGF | **-0.669** | **0.201** | **-3.336** | **0.001** |
| GluA4 | **0.518** | **0.232** | **2.231** | **0.027** | GluA4 | **-0.676** | **0.204** | **-3.309** | **0.001** |
| **Cerebellar FA** | Coeff | *SE* | *t* | *p* | **Cerebellar MD** | Coeff | *SE* | *t* | *p* |
| NPTX2 | 0.206 | 0.263 | 0.783 | 0.435 | NPTX2 | **-0.759** | **0.290** | **-2.619** | **0.010** |
| VGF | 0.451 | 0.239 | 1.886 | 0.061 | VGF | **-0.785** | **0.259** | **-3.028** | **0.003** |
| GluA4 | 0.377 | 0.245 | 1.540 | 0.126 | GluA4 | **-1.034** | **0.258** | **-4.012** | **<0.001** |

Note. **Bold**, un-corrected *p* < .05. Coeff = coefficient; FA = fractional anisotropy; MD = mean diffusivity; MTL = medial temporal lobe; SE = standard error.

**Predictor confidence intervals and model *R*^2^ values**

*Supplemental Table 7.* Predictor-level unstandardized beta values and 95% confidence intervals with model *R*^2^ values from multiple linear regression models assessing the associations of synaptic protein levels with white matter microstructure covarying age, sex, *APOE* ε4, diagnostic status.

| **Cerebral FA** | Predictor of Interest Coeff [95% CI] | Model *R*^2^ | **Cerebral MD** | Predictor of Interest Coeff  [95% CI] | Model *R*^2^ |
| --- | --- | --- | --- | --- | --- |
| NPTX2 | 0.34 [-0.14, 0.82] | 0.22 | NPTX2 | -0.41 [-0.87, 0.05] | 0.37 |
| VGF | **0.55 [0.11, 0.20]** | **0.23** | VGF | **-0.48 [-0.90, -0.06]** | **0.37** |
| GluA4 | **0.47 [0.01, 0.93]** | **0.22** | GluA4 | **-0.45 [-0.87, -0.03]** | **0.37** |
| **MTL FA** | Predictor of Interest Coeff [95% CI] | Model *R*^2^ | **MTL MD** | Predictor of Interest Coeff [95% CI] | Model *R*^2^ |
| NPTX2 | 0.33 [-0.16, 0.82] | 0.27 | NPTX2 | **-0.66 [-1.10, -0.23]** | **0.36** |
| VGF | **0.49 [0.04, 0.93]** | **0.27** | VGF | **-0.67 [-1.07, -0.28]** | **0.39** |
| GluA4 | **0.52 [0.06, 0.98]** | **0.28** | GluA4 | **-0.69 [-1.09, -0.28]** | **0.39** |
| **Cerebellar FA** | Predictor of Interest Coeff [95% CI] | Model *R*^2^ | **Cerebellar MD** | Predictor of Interest Coeff [95% CI] | Model *R*^2^ |
| NPTX2 | 0.21 [-0.31, 0.73] | 0.01 | NPTX2 | **-0.79 [-1.36, -0.22]** | **0.13** |
| VGF | 0.46 [-0.01, 0.93] | 0.03 | VGF | **-0.81 [-1.32, -0.30]** | **0.14** |
| GluA4 | 0.36 [-0.12, 0.85] | 0.02 | GluA4 | **-1.03 [-1.54, -0.52]** | **0.17** |

**Bold**, predictor un-corrected *p* < .05. Coeff = coefficient; FA = fractional anisotropy; MD = mean diffusivity; MTL = medial temporal lobe; SE = standard error.

*Supplemental Table 8.* Predictor-level unstandardized beta values and 95% confidence intervals with model *R*^2^ values from multiple linear regression models assessing the associations of white matter microstructure and synaptic proteins with executive function performance, covarying age, sex, and education.

| **Executive Function** | Predictor of Interest *β*  [95% CI] | Model *R*^2^ |
| --- | --- | --- |
| Cerebral FA | **0.06 [0.01, 0.12]** | **0.31** |
| Cerebral MD | **-0.07 [-0.13, -0.02]** | **0.32** |
| MTL FA | 0.05 [-0.01, 0.10] | 0.30 |
| MTL MD | **-0.06 [-0.12, -0.00]** | **0.31** |
| Cerebellar FA | **0.06 [0.012, 0.11]** | **0.31** |
| Cerebellar MD | **-0.05 [-0.09, -0.00]** | **0.31** |
| NPTX2 | 0.06 [-0.02, 0.14] | 0.29 |
| VGF | **0.07 [0.00, 0.14]** | **0.30** |
| GluA4 | 0.05 [-0.03, 0.12] | 0.29 |

**Bold**, predictor un-corrected *p* < .05. Coeff = coefficient; FA = fractional anisotropy; MD = mean diffusivity; MTL = medial temporal lobe; SE = standard error.

References

Gottesman, R. F., Albert, M. S., Alonso, A., Coker, L. H., Coresh, J., Davis, S. M., Deal, J. A., McKhann, G. M., Mosley, T. H., & Sharrett, A. R. (2017). Associations between midlife vascular risk factors and 25-year incident dementia in the Atherosclerosis Risk in Communities (ARIC) cohort. *JAMA neurology*, *74*(10), 1246-1254. <https://doi.org/10.1001/jamaneurol.2017.1658>

Pettigrew, C., Soldan, A., Wang, J., Wang, M.-C., Arthur, K., Moghekar, A., Gottesman, R. F., & Albert, M. (2020). Association of midlife vascular risk and AD biomarkers with subsequent cognitive decline. *Neurology*, *95*(23), e3093-e3103. <https://doi.org/10.1212/WNL.0000000000010946>

Soldan, A., Pettigrew, C., Zhu, Y., Wang, M. C., Gottesman, R. F., DeCarli, C., Albert, M., & Team, B. R. (2020). Cognitive reserve and midlife vascular risk: Cognitive and clinical outcomes. *Annals of Clinical and Translational Neurology*, *7*(8), 1307-1317. <https://doi.org/10.1002/acn3.51120>
